# Statistical design and analysis of controlled human malaria infection trials

**DOI:** 10.1186/s12936-024-04959-2

**Published:** 2024-05-03

**Authors:** Xiaowen Tian, Holly E. Janes, James G. Kublin

**Affiliations:** 1 Department of Biostatistics, University of Washington, 3980 15th Ave NE, Seattle, WA 98195 USA; 2 Vaccine and Infectious Disease Division, Fred Hutchinson Cancer Center, 1100 Fairview Ave N, Seattle, WA 98109 USA; 3 Department of Global Health, University of Washington, 3980 15th Ave NE, Seattle, WA 98195 USA

**Keywords:** Malaria, Challenge trial, Trial design

## Abstract

**Background:**

Malaria is a potentially life-threatening disease caused by *Plasmodium* protozoa transmitted by infected *Anopheles* mosquitoes. Controlled human malaria infection (CHMI) trials are used to assess the efficacy of interventions for malaria elimination. The operating characteristics of statistical methods for assessing the ability of interventions to protect individuals from malaria is uncertain in small CHMI studies. This paper presents simulation studies comparing the performance of a variety of statistical methods for assessing efficacy of intervention in CHMI trials.

**Methods:**

Two types of CHMI designs were investigated: the commonly used single high-dose design (SHD) and the repeated low-dose design (RLD), motivated by simian immunodeficiency virus (SIV) challenge studies. In the context of SHD, the primary efficacy endpoint is typically time to infection. Using a continuous time survival model, five statistical tests for assessing the extent to which an intervention confers partial or full protection under single dose CHMI designs were evaluated. For RLD, the primary efficacy endpoint is typically the binary infection status after a specific number of challenges. A discrete time survival model was used to study the characteristics of RLD versus SHD challenge studies.

**Results:**

In a SHD study with the continuous time survival model, log-rank test and t-test are the most powerful and provide more interpretable results than Wilcoxon rank-sum tests and Lachenbruch tests, while the likelihood ratio test is uniformly most powerful but requires knowledge of the underlying probability model. In the discrete time survival model setting, SHDs are more powerful for assessing the efficacy of an intervention to prevent infection than RLDs. However, additional information can be inferred from RLD challenge designs, particularly using a likelihood ratio test.

**Conclusions:**

Different statistical methods can be used to analyze controlled human malaria infection (CHMI) experiments, and the choice of method depends on the specific characteristics of the experiment, such as the sample size allocation between the control and intervention groups, and the nature of the intervention. The simulation results provide guidance for the trade off in statistical power when choosing between different statistical methods and study designs.

**Supplementary Information:**

The online version contains supplementary material available at 10.1186/s12936-024-04959-2.

## Background

Malaria is a potentially life-threatening disease caused by *Plasmodium* protozoa, which are transmitted by the bite of an infected female *Anopheles* mosquito. An estimated 241 million cases of malaria occurred worldwide in 2020 and there were an estimated 627,000 deaths from malaria globally [1]. While vector control, insecticide-treated mosquito nets, anti-malarial drugs, and indoor spraying with residual insecticides are powerful prevention tools that have helped to reduce disease burden, insecticide resistance and anti-malarial drug resistance are recurring problems [1]. The RTS,S/AS01 and R21-Matrix-M are the only vaccines thus far shown to confer protection from clinical malaria, albeit only partial protection that is short-lived and seems to depend on the level of background transmission [2–5]. There is active research into additional vaccine concepts and prophylactic drug regimens that will be needed for malaria elimination [6–8].

A controlled human malaria infection (CHMI) trial is a powerful tool for assessing the efficacy of a candidate intervention. A typical CHMI trial enrolls and randomizes healthy volunteers to control or intervention and exposes or “challenges” participants at a single point in time with infectious bites from laboratory-reared *Anopheles* mosquitoes carrying *Plasmodium falciparum* (*P. falciparum*) sporozoites, through needle injection of a defined number of aseptic cryopreserved sporozoites [9–12], or by the induced blood-stage model in which volunteers are inoculated with *P. falciparum*-infected erythrocytes [13]. Volunteers are typically followed for 28 days post-challenge [14] and tested for malaria infection using thick blood smear or quantitative PCR [9]. Most studies are designed such that all individuals in the control group are infected after this single challenge.

In the context of a CHMI trial, an intervention may influence malaria infection by offering either full protection or partial protection for a given individual. Fully protected individuals have negative malaria test results throughout follow-up, whereas partially protected individuals acquire infection but with delayed timing relative to the control group. Single challenge CHMI studies often use an infection indicator and time to test positivity as the primary efficacy outcomes of interest [14–17]. However, it is unknown which statistical methods best assess an intervention’s ability to partially and/or fully protect individuals from malaria. Classical methods for the analysis of binary outcomes, log-rank tests and tests of binomial infection probabilities, are commonly employed. However, it has not yet been established whether these have adequate statistical performance in the small sample sizes typical of CHMI studies, or whether there are better analysis methods or variations. With a continuous time survival model, simulation studies were used to evaluate the power of five statistical tests for assessing the extent to which an intervention confers partial or full protection in *P. falciparum* challenge studies. In doing so, software was developed for implementing these tests and for comparing their statistical performance under various design and simulation model parameter settings. This software is available through an publicly available R package and may be useful for future challenge study design.

A variety of statistical design and analysis methods have been developed to evaluate candidate HIV vaccines in simian immunodeficiency virus (SIV) challenge studies in nonhuman primates (NHPs). In particular, Regoes et al. and Hudgens and Gilbert have shown that repeated low dose challenge trial designs can be adequately powered to test for vaccine efficacy to prevent infection [18, 19]. Whereas a high-dose challenge study is designed to infect all or a high proportion of control participants, a ’low’ dose challenge is designed to infect only a fraction, and in so doing there is additional information generated as participants are repeatedly challenged. In the infectivity study described in Sheehy et al. [20], five out of six participants receiving 2500 sporozoites intradermally, three out of six participants receiving 2500 sporozoites intramuscularly and six out of six participants receiving 25,000 sporozoites intramuscularly were infected. While dose-ranging challenge trials in malaria have been conducted to identify the dose required to achieve 100% infection in the control group [11, 20–22], based on a review of the literature repeated low dose challenge studies have not been investigated for their utility in evaluating efficacy and mechanisms thereof. The statistical power of single high dose versus repeated low dose challenge study designs for evaluating efficacy in malaria or other human challenge studies was assessed using a discrete time survival model.

## Methods

### Continuous time survival model

For SHD, the primary efficacy endpoint is typically a continuous time-to-event variable, time to infection. This section describes using a continuous time survival model to evaluate the power of five statistical tests for assessing the extent to which an intervention confers partial or full protection under single dose CHMI designs. The model is motivated by collective data from recent CHMI studies. Reflecting the challenge models currently used in the field, it is assumed that the single challenge results in malaria infection in all control recipients and only consider designs with one intervention and a control group. In practice, it is not uncommon for studies to include intervention groups with different doses or intervention regimens which are compared to the control group individually or pooled together to assess efficacy [23, 24].

#### Simulation models

Sama et al. [25] suggested that Weibull distribution is a reasonable choice to approximate the lifespans of *P. falciparum* infections. The fit of Weibull distribution was examined using data from five previously published CHMI studies [26] and it was found that the Weibull distribution aligns well with the data. Additional file 1: Fig. S1 shows the quantile-quantile plot of Weibull distribution against data from five CHMI studies. The Weibull model was also fitted to data for 12 participants from two recent CHMI studies [27, 28] and the model fit the data well. Therefore, the Weibull distribution was used to simulate the time to infection.

Let $T_{01}, T_{02},..., T_{0n_0}$ and $T_{11}, T_{12},..., T_{1n_1}$ denote the times to test positivity for $n_0$ individuals in the control group and $n_1$ individuals in the intervention group, respectively. Times to test positivity for the control group, $T_{01}, T_{02},..., T_{0n_0}$, were simulated as i.i.d draws from the Weibull distributions with shape parameter *k* and scale parameter $\lambda _0$ such that the probability density function is of the form $f(T_{0i}=t;k,\lambda _0)=k\lambda _0^k t^{k-1}e^{-(\lambda _0 t)^k}$. Delays in time until test positivity conferred by the intervention, $T_{11}, T_{12},..., T_{1n_1}$, were simulated under i.i.d Weibull distributions with shape parameter *k* and scale parameter $\lambda _1$. Under this model, the delay in time to infection, i.e. partial protection, is parameterized by a reduction in the hazard rate in the intervention group. Let $\beta$ be the hazard ratio between the intervention and control groups, where $\beta =(\frac{\lambda _1}{\lambda _0})^k$ is constant in time.

A intervention is said to confer ‘full protection’ to an individual if that individual never becomes infected following challenge, and therefore the time to test positivity is not observed due to administrative censoring. To reflect full protection for some individuals in the intervention group, a Bernoulli variable with mean $\rho$ was used to indicate whether the individual is fully protected, in which case the value of $T_{1i}$ was set to be censored at the end of follow-up. Therefore, for individuals in the control group, the probability of being censored is $S(T_{0i};k,\lambda _0)$ where *S* is the survival function for the Weibull distribution with parameters *k* and $\lambda _0$. For individuals in the intervention group, the probability of being censored is $\rho +(1-\rho )S(T_{1i},k,\lambda _1)$. Note that CHMI studies generally do not incorporate censoring due to participant loss to follow-up, hence simulations assumed no loss to follow-up.

**Fig. 1:**
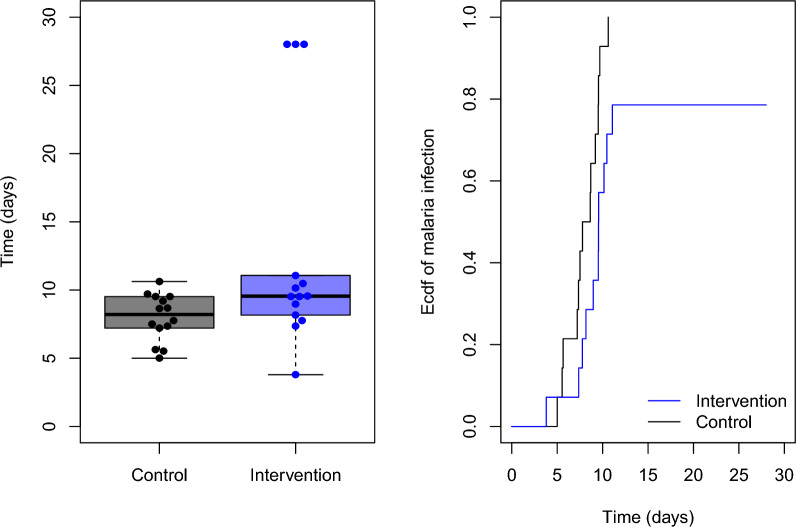
Example of simulated data for an intervention that confers full protection in 20% of participants in the intervention group and delays time to infection by 40% among those who become infected. Boxplots of the times to infection (left) and empirical cumulative infection probabilities (right) are shown for intervention and control groups. Fully protected individuals have a time of infection that is administratively censored at the end of follow-up (Day 28)

Also reflecting commonly-employed CHMI designs, the total sample size $N=n_0+n_1$ was set to be 16, 28, or 40 and the sample size allocation ratio $\frac{n_1}{n_0}$ to be 1 or 3. While equal allocation to control and intervention is likely optimal in most settings, allocating more participants to the intervention group has advantages for assessing secondary objectives such as evaluating safety and correlates of protection. Data from 12 placebo recipients from two recent CHMI trials [27, 28] were used to simulate time to test positivity (qRT-PCR$\ge$250) for the control group. Parameters *k* and $\lambda _0$ for the Weibull distribution were estimated by maximum likelihood. For the intervention group, the hazard ratio between the intervention group and control group, $\beta$, was set to take values from 0.2 to 1 with a step size of 0.2. Next, $\lambda _1$ was solved given $\beta ,k,\lambda _0$ and was used to simulate time to test positivity under a Weibull distribution with parameters *k* and $\lambda _1$. To incorporate the full protection effect in the intervention group, a Bernoulli random variable with mean $\rho$ was simulated to indicate whether an individual is fully protected, in which case the infection time was set to be censored at the end of follow-up (Day 28); $\rho$ takes values from 0 and 0.2. Note that if $\beta =1$ and $\rho =0$, the intervention group is simulated under the null model; if $\beta =1$ and $\rho >0$, the intervention effect consists only of full protection; and if $\beta <1$ and $\rho >0$, the intervention effect is a mixture of full and partial protection. Figure 1 shows an example of the simulated data under a mixture intervention effect. Table 1 shows the mean time to infection under the partial protection model.

**Table 1:** Mean time to infection based on simulation parameters under scenarios with $\rho = 0$

$\beta$	Mean time to infection in control group	Mean time to infection in intervention group
1.0	8.01	8.01
0.8	8.01	8.37
0.6	8.01	8.85
0.4	8.01	9.57
0.2	8.01	10.94

#### Statistical analysis

The power of five statistical tests for assessing an intervention’s efficacy was evaluated using Monte Carlo simulations. The two-sample t-test is used to compare the means of time to positivity between the control and intervention groups. The Wilcoxon rank-sum test is used to compare the distributional differences of time to positivity between the control and intervention groups. The log-rank test is used to compare the infection time distributions between the control and intervention groups. The Lachenbruch two part test compares the groups with respect to both the infection probability and the time to infection among those who become infected [29]. A parametric likelihood ratio test for detecting a mixture intervention effect was also investigated (see supplementary material). The likelihood ratio test was included as a reference as it is uniformly most powerful, but it requires knowledge of the underlying probability model. Therefore, the investigation focuses on which of the other tests is most powerful in small challenge-study settings.

### Discrete time survival model

For RLD, the primary efficacy endpoint is typically the infection status after a specific number of challenges. Therefore, a discrete time survival model [19] was used to study the characteristics of RLD and compare against SHD. Under this model, time is discrete and measured in number of challenges, whereas in the continuous time survival model time is measured in days since challenge. This section describes the evaluation of the power of the log-rank and likelihood ratio tests for assessing efficacy of an intervention under hypothetical SHD and RLD CHMI study designs.

#### Simulation models

Let $p_0$ and $p_1$ denote the probabilities that each of $n_0$ individuals in the control group and $n_1$ individuals in the intervention group become infected following a single challenge. Let $\rho$ be the probability that the intervention can fully protect an individual from the challenge. A strong assumption that the probability of infection is independent of the number of prior exposures was made. Hence, the probability of remaining uninfected following *t* challenges for an individual in the control group is $(1-p_0)^t$ and for an individual in the intervention group it is $(1-p_1)^t(1-\rho )+\rho$. The intervention efficacy was defined as the reduction in relative risk of infection per exposure, i.e. $1-\frac{p_1(1-\rho )}{p_0}$. If $\rho =0$, this is a ‘leaky model’ in the sense that all individuals will eventually become infected if followed long enough, and with $\rho >0$ it is a ‘mixture model’, as described in previous literature [19, 30, 31].

Let $c_{max}$ denote the maximum number of challenges for the trial. To mimic typical SHD challenge studies in which all individuals become infected following challenge [14, 16, 17, 32], $c_{max}=1$ and $p_0=1$ was set. To explore the RLD challenge design, variations were considered with $c_{max}$ set at 1, 3, 5 and $p_{0}$ set at 0.25, 0.5. Participants are challenged up to $c_{max}$ times until infection.

Again, the total sample size $N=n_0+n_1$ was set to be 16, 28, or 40 and the sample size allocation ratio $\frac{n_1}{n_0}$ to be 1 or 3. For the control arm, $p_0=1$ and $c_{max}=1$ were set for the SHD design and $p_0=0.25,0.5,0.75$ and $c_{max}=1,3,5$ for the RLD design. The relative infection probability $\frac{p_1}{p_0}$ takes values from 0.2 to 1 with a step size of 0.2. The full protection probability $\rho$ is 0 (leaky model) or 0.2 (mixture model). When $\frac{p_1}{p_0}=1$ and $\rho =0$, the data are simulated under the null hypothesis that the intervention has no effect. Additional file 1: Fig. S2 shows the simulated probability of remaining uninfected against the number of challenges under the leaky and mixture model.

#### Statistical analysis

Under the leaky model, the power of the log-rank and likelihood ratio tests for assessing the efficacy of the intervention was evaluated using Monte Carlo simulations. The log-rank test is used to assess whether there is a difference in the failure time (i.e. infection time) distribution between the control and intervention group, and the failure time is discrete and measured by the number of challenges until infection. Individuals who are not infected by the end of follow-up are censored at $c_{max}$ challenges. The likelihood ratio test, described in Hudgens and Gilbert [19], is used to test the null hypothesis that the intervention has no effect in reducing the per-challenge infection probability, i.e. $H_0: \frac{p_1}{p_0}=1$, against the alternative hypothesis that $\frac{p_1}{p_0}<1$. Under a SHD design and mixture model, only the power of the log-rank test was evaluated because the model is not identifiable for inferring both $\rho$ and $\frac{p_1}{p_0}$.

## Results

### Continuous time survival model

The operating characteristics of the five statistical tests for assessing an intervention’s efficacy described in the methods section were evaluated using 1000 Monte Carlo simulations. The comparisons with a total sample size of $N=28$ are shown in Fig. 3. Simulation results with $N=16,40$ are shown in Additional file 1: Fig. S3, S4. Type I error was assessed in scenarios under null hypothesis ($\rho =0$ and $\beta =1$). When $\rho =0$ and hazard ratio = 1. As expected, when there is no full protection effect ($\rho =0$), it is observed that the log-rank test is the most powerful test as the data are generated under the assumption of proportional hazards.

**Fig. 2:**
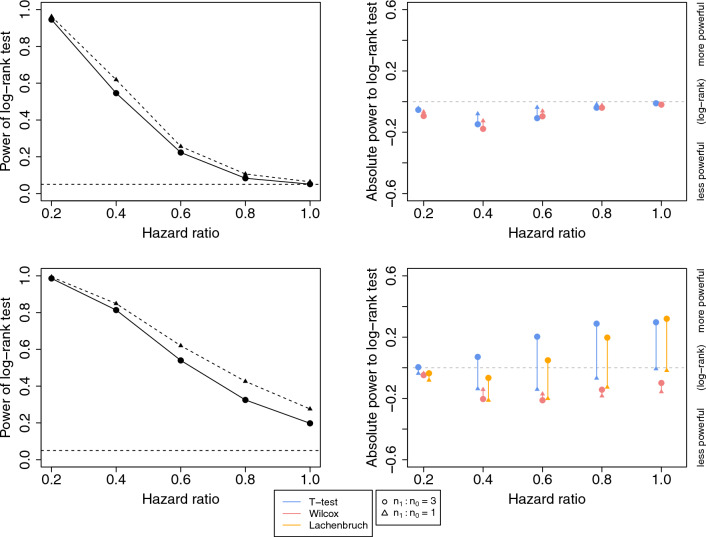
Power and type I error comparisons between log-rank, t-test, Wilcoxon, and Lachenbruch tests of differences between groups regarding time to malaria positivity. Results are based on simulations with total sample size $N=28$. The left panels demonstrate the power of the log-rank test with different sample size allocations. Triangles represent simulations with $\frac{n_1}{n_0}=1$ and circles represent simulations with $\frac{n_1}{n_0}=3$. The right panels demonstrate the absolute difference in power when comparing the t-test, Wilcoxon test, and Lachenbruch test to the log-rank test. The log-rank test is less powerful if the absolute power difference is negative. The top panels are based on simulations with no full protection effect ($\rho =0$), with hazard ratio = 1, the points represent the type I error. The bottom panels are based on simulations with 20% full protection ($\rho =0.2$)

When the intervention effect is a mixture of partial and full protection, the mixture likelihood ratio test is the most powerful test since the data are generated under the mixture model and the proportional hazards assumption no longer holds. However, the likelihood ratio test relies heavily on the assumed failure time distribution and will become less reliable when the assumption is violated. Comparing the performance of the tests that do not make this strong distributional assumption, the difference in power between the t-test and the log-rank test varies with the sample size allocation between the control and intervention groups. This is because the optimal sample size allocation for the t-test depends on the ratio of the variance in the time to infection between the two groups. For example, with 20% full protection ($\rho =0.2$) and a 40% reduction in hazard of infection ($\beta =0.6$), the log-rank test is 15% more powerful than the t-test (absolute difference in power is 15%) when $\frac{n_1}{n_0}=1$. However, when $\frac{n_1}{n_0}=3$, the t-test is 20% more powerful than the log-rank test (see Fig. 2). To help explain this phenomenon, assume that the variance is known and the z-test is used instead of the t-test. The variance of the z-test statistic is minimized when the ratio of the variance between the intervention and control groups is equal to the square of the ratio of the sample sizes between the intervention and control groups. Indeed, Additional file 1: Fig. S5 shows that the power of the t-test is highest when $\frac{n_1}{n_0}=3$, which is approximately the ratio of standard deviations in the intervention and control groups under $\rho =0.2$. Under the assumption of no loss to follow-up, time to positivity in the intervention group was truncated at 28 days, hence the t-test should be interpreted as a test of a difference in truncated means and should not be used when the assumption of no loss to follow-up is violated. Given the concerns about small-sample performance of asymptotic-based tests, randomization-based tests, i.e. permutation tests, were also evaluated (Additional file 1: Fig. S6). It is generally found that asymptotic-based tests are slightly more powerful than permutation tests.

The Lachenbruch test statistic is a linear combination of the Wilcoxon rank-sum test conditional on infection status and a binomial test of infection rates. Based on our simulation scheme, it is possible to observe that none of the individuals in the intervention group are fully protected by chance for some values of $\rho$. In this case, the Lachenbruch test reduces to the Wilcoxon test. From the simulation results, the power of the Lachenbruch test is comparable to that of the t-test and both tests tend to be more powerful compared to the log-rank test as the sample size increases. However, that the Wilcoxon rank-sum test conditional on infection status breaks the randomization. Therefore, in practice the statistical inference may be confounded by other variables that differ between infected individuals in the two treatment groups, i.e. it is subject to post-randomization selection bias [33, 34]. For this reason, its use in general practice is not advocated.

### Discrete time survival model

**Fig. 3:**
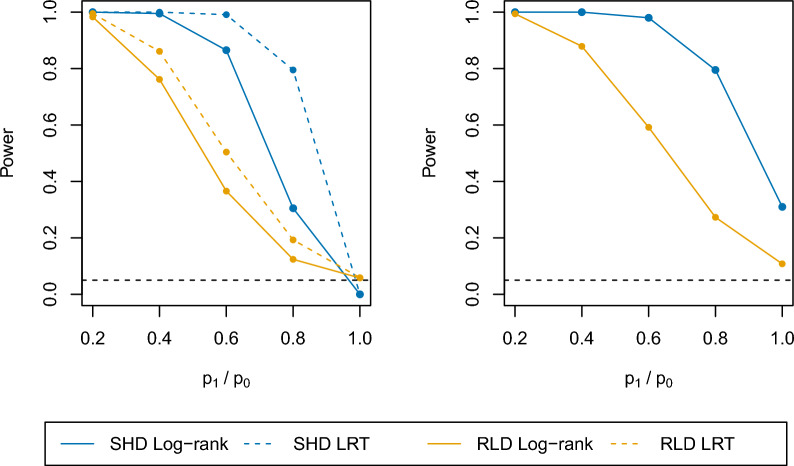
Power and type I error comparisons between single high-dose and repeated low-dose challenge study designs. The results are based on simulations with $n_0=n_1=14, p_0=1$ for the SHD design, $p_0=0.5$ and $c_{max}=5$ for the RLD design. The left panel demonstrates the scenario without full protection effect ($\rho =0$) where both log-rank and likelihood-ratio tests are evaluated. The right panel demonstrates the scenario with 20% full protection ($\rho =0.2$) and only log-rank test is evaluated With $p_1/p_0=1$ and $\rho =0$, the points represent the type I error

The operating characteristics of the log-rank test and likelihood ratio test described in the methods section were evaluated using 1000 Monte Carlo simulations. As shown in Additional file 1: Table S1, the power of the log-rank and likelihood ratio tests increases as a function of the maximum number of challenges for a RLD design. Therefore, to compare the SHD and RLD designs, the focus is on on $c_{max}=1$ for the SHD design and $c_{max}=5$ for the RLD design. The power and type I error comparisons based on a total sample size of $N=28$ and $\frac{n_1}{n_0}=1$ are shown in Fig. 3. It is observed that statistical tests in the setting of a SHD design tend to be conservative when intervention efficacy is low. This is due to the boundary problem that when $p_0=1, \rho =0$ and $p_1$ is close to 1, all simulated participants may become infected by chance and there is no variability in the dataset. When comparing a SHD design to a RLD design, both the log-rank and likelihood ratio tests are found to have greater statistical power regardless of whether the intervention is leaky or a mixture model. This observation is consistent across different values of $N, \frac{n_1}{n_0}$, and $p_0$ (see supplementary material). Thus, the results demonstrate that the SHD challenge design is superior to the RLD challenge design in terms of statistical power for our simulation settings. However, while the RLD challenge design has lower statistical power as compared to SHD design, it has advantages for the study of immune correlates and for further understanding the induction of natural immunity [35, 36]. These results could provide a guidance for the trade off in statistical power when choosing a RLD design over a SHD design.

## Discussion

A variety of statistical methods can be applied for analyzing controlled human malaria infection (CHMI) studies. A continuous time survival model was used to evaluate and compare the power of t-tests, log-rank tests, Wilcoxon rank-sum tests, Lachenbruch tests, and likelihood ratio tests in *P. falciparum* challenge studies. While the likelihood ratio test is uniformly most powerful, it requires knowledge of the underlying probability model. Comparing the performance of the tests not relying on strong model assumptions, it is found that the log-rank test and t-test are the most powerful. Importantly, these tests also provide more interpretable results than do the Wilcoxon rank-sum and Lachenbruch tests. The difference in power between the t- and log-rank tests varies with the sample size allocation between the control and intervention groups, due to the fact that the optimal power for the t-test depends on the ratio of variance and the ratio of sample size between two groups. It is found that the type I error rate is appropriately controlled for asymptotic-based tests even with the small sample sizes typical of CHMI studies. The simulations assumed the only censoring mechanism is administrative censoring at the end of the study. Censoring for loss to follow-up can be accommodated under the some of the analytic methods that were investigated here, such as the log-rank test and the likelihood ratio test, however, the impact of loss to follow-up was not investigated in the simulation studies since CHMI studies generally do not incorporate censoring due to participants loss to follow-up.

This simulation study focused on the infection status and time to infection hence may not encapsulate the complete range of complexities that are characteristics of the CHMI design such as the frequency of sample collection and variations in parasite kinetics. While these factors also play an important role in CHMI studies, they were not within the scope of the investigation. Further research that explore these impacts could provide a more comprehensive understanding of CHMI studies.

Motivated by the RLD for simian immunodeficiency virus (SIV) challenge studies in nonhuman primates, whether RLDs are superior to SHDs in the context of CHMI experiments was also investigated. A discrete time survival model was used to compare the power of log-rank and likelihood ratio tests in different simulation settings. It is found that SHDs are more powerful for assessing the efficacy of an intervention to prevent infection. However, it should be noted that additional information can be inferred from RLD challenge designs. In particular, using a likelihood ratio test, a partial intervention effect as measured by a reduction in the per-challenge infection probability can be distinguished from a full protection effect. This is under the assumption that the infection probability varies with the number of challenges while the full protection effect is time-invariant.

There are many possible future study directions regarding design and analysis of RLD challenge studies in the context of malaria. The discrete time simulation studies and all prior methodology for repeated challenge designs assume that the malaria infection probability is the same across individuals and that it does not vary with the number of challenges. This is an oversimplification of the natural infection mechanism and immunological learning that occurs following exposure. Hudgens and Gilbert [19] suggested using a beta distribution model for the infection probability such that the control and intervention groups are assumed to have the same coefficient of variation but possibly different means. Extending this or a related model to accommodate an infection probability that varies with the number of challenges is a topic for future research.

The comparison of different statistical tests is based on simulated data where the response is homogeneous within the control group and heterogeneous within the intervention group. The parameters used for the simulations are based on malaria challenge trials and therefore reflect data for this setting. Whether the conclusions can be generalized to other pathogens such as dengue and influenza warrants further investigation.

A variation on the above RLD design would be to re-challenge all participants, even those who become infected after a given challenge, up to a maximum number of challenges. This design has the advantage that it would potentially allow one to evaluate how both full and partial protection vary as a function of exposure and infection history, and thus provide new insights into mechanism of action and correlates of protection. Such designs have been used in challenge studies for malaria [35], *Shigella* [37], tuberculosis [38], norovirus [39], pneumococcus [40], and Enterotoxigenic *Escherichia coli* (ETEC) [41]. While most of these studies aimed to investigate the induction of natural immunity due to previous exposure, few placed emphasis on evaluation of vaccine efficacy. Transition models [42] may be useful for modelling the dependence of infection status on previous exposure and infection history. Alternatively, ordinary logistic regression or generalized estimating equations [42–44] may be used to do inference conditional on a given exposure and infection history, or marginalized transition models [45] may be used to evaluate the marginalized effect of intervention and provide consistent estimates even if the dependence model is mis-specified. The optimal statistical framework and inferential approach deserve further investigation. As well, the feasibility of RLD malaria challenge trials needs to be explored. These investigations and advancements in modeling, immunology, and clinical research will contribute towards the ultimate goal of malaria elimination.

### Supplementary Information

**Additional file 1: Table S1.** Power analysis for RLD. The results are based on simulations with* N* = 28,* ρ* = 0,* p*_0_ = 0.25. **Table S2.** Power analysis for RLD. The results are based on simulations with * N* = 28, * ρ* = 0,* p*_0_ = 0.5. **Table S3.** Power analysis for RLD. The results are based on simulations with N = 28, * ρ* = 0,* p*_0_ = 0.75.** Table S4.** Power analysis for SHD. The results are based on simulations with * N* = 28, *ρ* = 0, *p*_0_ = 1. **Table S5.** Power analysis for RLD. The results are based on simulations with* N* = 16, * ρ* = 0, *p*_0_ = 0.25. **Table S6.** Power analysis for RLD. The results are based on simulations with * N* = 16, * ρ* = 0,* p*_0_ = 0.5. **Table S7.** Power analysis for RLD. The results are based on simulations with *N *= 16, *ρ *= 0*,* *p*_0_ = 0*.*75. **Table S8.** Power analysis for SHD. The results are based on simulations with *N *= 16*,* *ρ* = 0*, p*_0_ = 1. **Table S9.** Power analysis for RLD. The results are based on simulations with *N *= 40*,* *ρ* = 0*,* *p*_0_ = 0*.*25. **Table S10.** Power analysis for RLD. The results are based on simulations with *N *= 40*, ρ *= 0*,* *p*_0_ = 0*.*5. **Table S11.** Power analysis for RLD. The results are based on simulations with *N *= 40*, ρ *= 0*, p*_0_ = 0*.*75. **Table S12.** Power analysis for SHD. The results are based on simulations with *N *= 40*,* *ρ* = 0*,* *p*_0_ = 1. **Figure S1.** Quantile-quantile plot of time to infection from 5 previously published CHMI studies. **Figure S2.** Probability of remaining uninfected against the number of challenges under leaky (*ρ *= 0) and mixture model (*ρ *= 0.2). *p*_1_ and *p*_0_ represent the probability of becoming infected after a single challenge in the intervention and control group, respectively. **Figure S3.** Power and type I error comparisons: Results are based on simulations with *N *= 16. The upper panels demonstrate the power of the log-rank test with different sample size allocations. Triangles represent simulations with $\frac{{n_{1} }}{{n_{0} }}$= 1 and circles represent simulations with $\frac{{n_{1} }}{{n_{0} }}$ = 3. The lower panels demonstrate the absolute difference in power when comparing the t-test, Wilcoxon test, and Lachenbruch test to the log-rank test. The log-rank test is less powerful if the absolute power of the log-rank test is negative. **Figure S4.** Power and type I error comparisons: Results are based on simulations with *N *= 40. The upper panels demonstrate the power of the log-rank test with different sample size allocations. Triangles represent simulations with $\frac{{n_{1} }}{{n_{0} }}$= 1 and circles represent simulations with $\frac{{n_{1} }}{{n_{0} }}$ = 3. The lower panels demonstrate the absolute difference in power when comparing the t-test, Wilcoxon test, and Lachenbruch test to the log-rank test. The log-rank test is less powerful if the absolute power of the log-rank test is negative. **Figure S5.** Power of the t-test for different sample size allocations: Results are based on simulations with *ρ *= 0*.*2. The t-test is most powerful when $\frac{{n_{1} }}{{n_{0} }}$= 3. The square root of the ratio of variance between the intervention and control groups in the simulated data ranges from 4.2 to 4.6 for *β *∈ [0*.*2*,*1]. **Figure S6.** Power and type I error comparisons of asymptotic-based and permutation-based tests. Results are shown for different sample size allocations and for *ρ *= 0 and *ρ *= 0*.*2. The total sample size is fixed at 28. **Figure S7.** Power and type I error comparisons. The results are based on simulations with *n*_0_ = *n*_1_ = 8*,* *p*_0_ = 1 for SHD, *p*_0_ = 0*.*5 and *c*_*max*_ = 5 for RLD. With relative risk being 1, the points represent the type I error. **Figure S8.** Power and type I error comparisons. The results are based on simulations with *n*_0_ = *n*_1_ = 20*, p*_0_ = 1 for SHD, *p*_0_ = 0*.*5 and *c*_*max*_ = 5 for RLD. With relative risk being 1, the points represent the type I error.

## Data Availability

All data generated or analysed during this study are included in this published article (and its additional information files). The R package for power and sample size calculation is available on https://github.com/tianxiaowen/CHMI.

## References

[1] World Health Organization (2021). World Malaria Report 2021.

[2] RTS, S Clinical Trials Partnership (2015). Efficacy and safety of RTS, S/AS01 malaria vaccine with or without a booster dose in infants and children in Africa: final results of a phase 3, individually randomised, controlled trial. Lancet.

[3] Agnandji ST, Fernandes JF, Bache EB, Ramharter M (2015). Clinical development of RTS, S/AS malaria vaccine: a systematic review of clinical Phase I-III trials. Future Microbiol.

[4] Zavala F (2022). RTS, S: the first malaria vaccine. J Clin Invest.

[5] Datoo MS, Natama MH, Somé A, Traoré O, Rouamba T, Bellamy D (2021). Efficacy of a low-dose candidate malaria vaccine, R21 in adjuvant Matrix-M, with seasonal administration to children in Burkina Faso: a randomised controlled trial. Lancet.

[6] Hoffman SL, Vekemans J, Richie TL, Duffy PE (2015). The march toward malaria vaccines. Vaccine.

[7] Draper SJ, Sack BK, King CR, Nielsen CM, Rayner JC, Higgins MK (2018). Malaria vaccines: recent advances and new horizons. Cell Host Microbe.

[8] Ashley EA, Phyo AP (2018). Drugs in development for malaria. Drugs.

[9] Cooper MM, Loiseau C, McCarthy JS, Doolan DL (2019). Human challenge models: tools to accelerate the development of malaria vaccines. Expert Rev Vaccines.

[10] Hoffman SL, Goh LM, Luke TC, Schneider I, Le TP, Doolan DL (2002). Protection of humans against malaria by immunization with radiation-attenuated Plasmodium falciparum sporozoites. J Infect Dis.

[11] Roestenberg M, Bijker EM, Sim BKL, Billingsley PF, James ER, Bastiaens GJH (2013). Controlled human malaria infections by intradermal injection of cryopreserved Plasmodium falciparum sporozoites. Am J Trop Med Hyg.

[12] Gomez-Perez GP, Legarda A, Munoz J, Sim BK, Ballester MR, Dobano C (2015). Controlled human malaria infection by intramuscular and direct venous inoculation of cryopreserved Plasmodium falciparum sporozoites in malaria-naïve volunteers: effect of injection volume and dose on infectivity rates. Malar J.

[13] McCarthy JS, Sekuloski S, Griffin PM, Elliott S, Douglas N, Peatey C (2011). A pilot randomised trial of induced blood-stage Plasmodium falciparum infections in healthy volunteers for testing efficacy of new antimalarial drugs. PLoS ONE.

[14] Murphy SC, Duke ER, Shipman KJ, Jensen RL, Fong Y, Ferguson S (2018). A randomized trial evaluating the prophylactic activity of DSM265 against preerythrocytic Plasmodium falciparum infection during controlled human malarial infection by mosquito bites and direct venous inoculation. J Infect Dis.

[15] Bastiaens GJH, van Meer MPA, Scholzen A, Obiero JM, Vatanshenassan M, van Grinsven T (2016). Safety, immunogenicity, and protective efficacy of intradermal immunization with aseptic, purified, cryopreserved Plasmodium falciparum sporozoites in volunteers under chloroquine prophylaxis: a randomized controlled trial. Am J Trop Med Hyg.

[16] Sulyok M, Ruckle T, Roth A, Murbeth RE, Chalon S, Kerr N (2017). DSM265 for *Plasmodium falciparum* chemoprophylaxis: a randomised, double blinded, phase 1 trial with controlled human malaria infection. Lancet Infect Dis.

[17] McCarthy JS, Lotharius J, Ruckle T, Chalon S, Phillips MA, Elliott S (2017). Safety, tolerability, pharmacokinetics, and activity of the novel long-acting antimalarial DSM265: a two-part first-in-human phase 1a/1b randomised study. Lancet Infect Dis.

[18] Regoes RR, Longini IM, Feinberg MB, Staprans SI (2005). Preclinical assessment of HIV vaccines and microbicides by repeated low-dose virus challenges. PLoS Med.

[19] Hudgens MG, Gilbert PB (2009). Assessing vaccine effects in repeated low-dose challenge experiments. Biometrics.

[20] Sheehy SH, Spencer AJ, Douglas AD, Sim BKL, Longley RJ, Edwards NJ (2013). Optimising controlled human malaria infection studies using cryopreserved *P. falciparum* parasites administered by needle and syringe. PLoS ONE.

[21] Lyke KE, Laurens MB, Strauss K, Adams M, Billingsley PF, James E (2015). Optimizing intradermal administration of cryopreserved Plasmodium falciparum sporozoites in controlled human malaria infection. Am J Trop Med Hyg.

[22] Murphy SC, Vaughan AM, Kublin JG, Fishbauger M, Seilie AM, Cruz KP (2022). A genetically engineered Plasmodium falciparum parasite vaccine provides protection from controlled human malaria infection. Sci Transl Med.

[23] Kublin JG, Murphy SC, Maenza J, Seilie AM, Jain JP, Berger D (2021). Safety, pharmacokinetics, and causal prophylactic efficacy of KAF156 in a Plasmodium falciparum human infection study. Clin Infect Dis.

[24] von Borstel A, Chevour P, Arsovski D, Krol JM, Howson LJ, Berry AA (2021). Repeated *Plasmodium falciparum* infection in humans drives the clonal expansion of an adaptive γδ T cell repertoire. Sci Transl Med.

[25] Sama W, Dietz K, Smith T (2006). Distribution of survival times of deliberate *Plasmodium falciparum* infections in tertiary syphilis patients. Trans R Soc Trop Med Hyg.

[26] Coffeng LE, Hermsen CC, Sauerwein RW, de Vlas SJ (2017). The power of malaria vaccine trials using controlled human malaria infection. PLoS Comput Biol.

[27] Talley AK, Healy SA, Finney OC, Murphy SC, Kublin J, Salas CJ (2014). Safety and comparability of controlled human *Plasmodium falciparum* infection by mosquito bite in malaria-naïve subjects at a new facility for sporozoite challenge. PLoS ONE.

[28] Healy SA, Murphy SC, Hume JCC, Shelton L, Kuntz S, Van Voorhis WC (2019). Chemoprophylaxis vaccination: phase I study to explore stage-specific immunity to *Plasmodium falciparum* in US adults. Clin Infect Dis.

[29] Lachenbruch PA (2002). Analysis of data with excess zeros. Stat Methods Med Res.

[30] Halloran ME, Haber M, Longini J, Ira M (1992). Interpretation and estimation of vaccine efficacy under heterogeneity. Am J Epidemiol.

[31] Longini IM, Halloran ME (1996). A frailty mixture model for estimating vaccine efficacy. J R Stat Soc Ser C Appl Stat.

[32] Talley AK, Healy SA, Finney OC, Murphy SC, Kublin J, Salas CJ (2014). Safety and comparability of controlled human *Plasmodium falciparum* infection by mosquito bite in malaria-naïve subjects at a new facility for sporozoite challenge. PLoS ONE.

[33] Gilbert PB, Bosch RJ, Hudgens MG (2003). Sensitivity analysis for the assessment of causal vaccine effects on viral load in HIV vaccine trials. Biometrics.

[34] Mehrotra DV, Li X, Gilbert PB (2006). A comparison of eight methods for the dual-endpoint evaluation of efficacy in a proof-of-concept HIV vaccine trial. Biometrics.

[35] Pombo DJ, Lawrence G, Hirunpetcharat C, Rzepczyk C, Bryden M, Cloonan N (2002). Immunity to malaria after administration of ultra-low doses of red cells infected with *Plasmodium falciparum*. Lancet.

[36] Roestenberg M, Hoogerwerf MA, Ferreira DM, Mordmüller B, Yazdanbakhsh M (2018). Experimental infection of human volunteers. Lancet Infect Dis.

[37] Porter C, Thura N, Ranallo R, Riddle M (2013). The Shigella human challenge model. Epidemiol Infect.

[38] Minassian AM, Satti I, Poulton ID, Meyer J, Hill AVS, McShane H (2012). A human challenge model for *Mycobacterium tuberculosis* using Mycobacterium bovis bacille Calmette-Guerin. J Infect Dis.

[39] Parrino TA, Schreiber DS, Trier JS, Kapikian AZ, Blacklow NR (1977). Clinical immunity in acute gastroenteritis caused by norwalk agent. N Engl J Med.

[40] Ferreira DM, Neill DR, Bangert M, Gritzfeld JF, Green N, Wright AKA (2013). Controlled human infection and rechallenge with *Streptococcus pneumoniae* reveals the protective efficacy of carriage in healthy adults. Am J Respir Crit Care Med.

[41] Levine MM, Nalin DR, Hoover DL, Bergquist EJ, Hornick RB, Young CR (1979). Immunity to enterotoxigenic *Escherichia coli*. Infect Immun.

[42] Diggle P, Liang KY, Zeger SL (1994). Analysis of longitudinal data, Oxford statistical science series..

[43] Liang KY, Zeger SL (1986). Longitudinal data analysis using generalized linear models. Biometrika.

[44] Bible J, Albert PS, Simons-Morton BG, Liu D (2019). Practical issues in using generalized estimating equations for inference on transitions in longitudinal data: What is being estimated?. Stat Med.

[45] Heagerty PJ (2002). Marginalized transition models and likelihood inference for longitudinal categorical data. Biometrics.

