# Supplementary material for: Statistical design and analysis of controlled human malaria infection trials
Source: Malar J. 2024 May 3;23:133. doi: 10.1186/s12936-024-04959-2 (PMC11068571; doi:10.1186/s12936-024-04959-2)
Supplement: Supplementary file 1 — Additional file 1: Table S1. Power analysis for RLD. The results are based on simulations with N = 28, ρ = 0, p0 = 0.25. Table S2. Power analysis for RLD. The results are based on simulations with N = 28, ρ = 0, p0 = 0.5. Table S3. Power analysis for RLD. The results are based on simulations with N = 28, ρ = 0, p0 = 0.75. Table S4. Power analysis for SHD. The results are based on simulations with N = 28, ρ = 0, p0 = 1. Table S5. Power analysis for RLD. The results are based on simulations with N = 16, ρ = 0, p0 = 0.25. Table S6. Power analysis for RLD. The results are based on simulations with N = 16, ρ = 0, p0 = 0.5. Table S7. Power analysis for RLD. The results are based on simulations with N = 16, ρ = 0, p0 = 0.75. Table S8. Power analysis for SHD. The results are based on simulations with N = 16, ρ = 0, p0 = 1. Table S9. Power analysis for RLD. The results are based on simulations with N = 40, ρ = 0, p0 = 0.25. Table S10. Power analysis for RLD. The results are based on simulations with N = 40, ρ = 0, p0 = 0.5. Table S11. Power analysis for RLD. The results are based on simulations with N = 40, ρ = 0, p0 = 0.75. Table S12. Power analysis for SHD. The results are based on simulations with N = 40, ρ = 0, p0 = 1. Figure S1. Quantile-quantile plot of time to infection from 5 previously published CHMI studies. Figure S2. Probability of remaining uninfected against the number of challenges under leaky (ρ = 0) and mixture model (ρ = 0.2). p1 and p0 represent the probability of becoming infected after a single challenge in the intervention and control group, respectively. Figure S3. Power and type I error comparisons: Results are based on simulations with N = 16. The upper panels demonstrate the power of the log-rank test with different sample size allocations. Triangles represent simulations with [file 12936_2024_4959_MOESM1_ESM.pdf]

## Supplementary Information

| $\frac{p_1}{p_0}$ | $c_{max}$ | Log-rank($\frac{n_1}{n_0}=1$) | Log-rank($\frac{n_1}{n_0}=3$) | LRT($\frac{n_1}{n_0}=1$) | LRT($\frac{n_1}{n_0}=3$) |
|---|---|---|---|---|---|
| 1.0 | 1 | 0.051 | 0.044 | 0.069 | 0.051 |
| 1.0 | 3 | 0.047 | 0.046 | 0.044 | 0.055 |
| 1.0 | 5 | 0.063 | 0.058 | 0.055 | 0.060 |
| 0.8 | 1 | 0.053 | 0.043 | 0.107 | 0.092 |
| 0.8 | 3 | 0.094 | 0.096 | 0.134 | 0.126 |
| 0.8 | 5 | 0.086 | 0.085 | 0.145 | 0.134 |
| 0.6 | 1 | 0.108 | 0.089 | 0.202 | 0.155 |
| 0.6 | 3 | 0.208 | 0.158 | 0.319 | 0.233 |
| 0.6 | 5 | 0.236 | 0.212 | 0.358 | 0.284 |
| 0.4 | 1 | 0.159 | 0.139 | 0.334 | 0.232 |
| 0.4 | 3 | 0.365 | 0.360 | 0.532 | 0.446 |
| 0.4 | 5 | 0.518 | 0.439 | 0.653 | 0.535 |
| 0.2 | 1 | 0.274 | 0.298 | 0.549 | 0.444 |
| 0.2 | 3 | 0.725 | 0.674 | 0.847 | 0.744 |
| 0.2 | 5 | 0.871 | 0.813 | 0.937 | 0.855 |

Table S1: Power analysis for RLD. The results are based on simulations with $N = 28, \rho = 0, p_0 = 0.25$.

| $\frac{p_1}{p_0}$ | $c_{max}$ | Log-rank($\frac{n_1}{n_0}=1$) | Log-rank($\frac{n_1}{n_0}=3$) | LRT($\frac{n_1}{n_0}=1$) | LRT($\frac{n_1}{n_0}=3$) |
|---|---|---|---|---|---|
| 1.0 | 1 | 0.033 | 0.050 | 0.042 | 0.057 |
| 1.0 | 3 | 0.050 | 0.063 | 0.060 | 0.073 |
| 1.0 | 5 | 0.058 | 0.051 | 0.059 | 0.058 |
| 0.8 | 1 | 0.056 | 0.065 | 0.107 | 0.117 |
| 0.8 | 3 | 0.125 | 0.108 | 0.200 | 0.172 |
| 0.8 | 5 | 0.124 | 0.108 | 0.193 | 0.170 |
| 0.6 | 1 | 0.161 | 0.169 | 0.269 | 0.241 |
| 0.6 | 3 | 0.319 | 0.251 | 0.471 | 0.367 |
| 0.6 | 5 | 0.366 | 0.284 | 0.504 | 0.405 |
| 0.4 | 1 | 0.386 | 0.329 | 0.535 | 0.425 |
| 0.4 | 3 | 0.670 | 0.584 | 0.778 | 0.695 |
| 0.4 | 5 | 0.762 | 0.691 | 0.861 | 0.805 |
| 0.2 | 1 | 0.662 | 0.594 | 0.794 | 0.696 |
| 0.2 | 3 | 0.948 | 0.914 | 0.981 | 0.956 |
| 0.2 | 5 | 0.983 | 0.960 | 0.995 | 0.983 |

Table S2: Power analysis for RLD. The results are based on simulations with $N = 28, \rho = 0, p_0 = 0.5$.

| $\frac{p_1}{p_0}$ | $c_{max}$ | Log-rank($\frac{n_1}{n_0} = 1$) | Log-rank($\frac{n_1}{n_0} = 3$) | LRT($\frac{n_1}{n_0} = 1$) | LRT($\frac{n_1}{n_0} = 3$) |
|---|---|---|---|---|---|
| 1.0 | 1 | 0.059 | 0.040 | 0.079 | 0.085 |
| 1.0 | 3 | 0.034 | 0.040 | 0.046 | 0.092 |
| 1.0 | 5 | 0.052 | 0.046 | 0.063 | 0.099 |
| 0.8 | 1 | 0.139 | 0.074 | 0.230 | 0.216 |
| 0.8 | 3 | 0.177 | 0.089 | 0.285 | 0.221 |
| 0.8 | 5 | 0.163 | 0.099 | 0.292 | 0.242 |
| 0.6 | 1 | 0.355 | 0.271 | 0.500 | 0.440 |
| 0.6 | 3 | 0.491 | 0.345 | 0.659 | 0.552 |
| 0.6 | 5 | 0.532 | 0.368 | 0.689 | 0.542 |
| 0.4 | 1 | 0.664 | 0.585 | 0.787 | 0.720 |
| 0.4 | 3 | 0.897 | 0.777 | 0.948 | 0.894 |
| 0.4 | 5 | 0.908 | 0.783 | 0.973 | 0.904 |
| 0.2 | 1 | 0.939 | 0.881 | 0.973 | 0.928 |
| 0.2 | 3 | 0.998 | 0.991 | 1.000 | 0.996 |
| 0.2 | 5 | 0.999 | 0.992 | 1.000 | 0.999 |

Table S3: Power analysis for RLD. The results are based on simulations with $N = 28, \rho = 0, p_0 = 0.75$.

| $\frac{p_1}{p_0}$ | Log-rank($\frac{n_1}{n_0} = 1$) | Log-rank($\frac{n_1}{n_0} = 3$) | LRT($\frac{n_1}{n_0} = 1$) | LRT($\frac{n_1}{n_0} = 3$) |
|---|---|---|---|---|
| 1.0 | 0.000 | 0.000 | 0.000 | 0.000 |
| 0.8 | 0.305 | 0.008 | 0.795 | 0.438 |
| 0.6 | 0.865 | 0.445 | 0.991 | 0.962 |
| 0.4 | 0.995 | 0.959 | 1.000 | 1.000 |
| 0.2 | 1.000 | 1.000 | 1.000 | 1.000 |

Table S4: Power analysis for SHD. The results are based on simulations with $N = 28, \rho = 0, p_0 = 1$.

| $\frac{p_1}{p_0}$ | $c_{max}$ | Log-rank($\frac{n_1}{n_0}=1$) | Log-rank($\frac{n_1}{n_0}=3$) | LRT($\frac{n_1}{n_0}=1$) | LRT($\frac{n_1}{n_0}=3$) |
|---|---|---|---|---|---|
| 1.0 | 1 | 0.041 | 0.035 | 0.090 | 0.083 |
| 1.0 | 3 | 0.049 | 0.059 | 0.059 | 0.059 |
| 1.0 | 5 | 0.063 | 0.067 | 0.054 | 0.070 |
| 0.8 | 1 | 0.040 | 0.051 | 0.133 | 0.130 |
| 0.8 | 3 | 0.070 | 0.085 | 0.106 | 0.114 |
| 0.8 | 5 | 0.087 | 0.089 | 0.129 | 0.121 |
| 0.6 | 1 | 0.040 | 0.054 | 0.213 | 0.202 |
| 0.6 | 3 | 0.142 | 0.117 | 0.232 | 0.174 |
| 0.6 | 5 | 0.151 | 0.187 | 0.256 | 0.248 |
| 0.4 | 1 | 0.067 | 0.104 | 0.304 | 0.324 |
| 0.4 | 3 | 0.236 | 0.268 | 0.396 | 0.341 |
| 0.4 | 5 | 0.333 | 0.302 | 0.472 | 0.398 |
| 0.2 | 1 | 0.078 | 0.138 | 0.438 | 0.462 |
| 0.2 | 3 | 0.443 | 0.461 | 0.652 | 0.572 |
| 0.2 | 5 | 0.612 | 0.608 | 0.751 | 0.689 |

Table S5: Power analysis for RLD. The results are based on simulations with $N = 16, \rho = 0, p_0 = 0.25$.

| $\frac{p_1}{p_0}$ | $c_{max}$ | Log-rank($\frac{n_1}{n_0} = 1$) | Log-rank($\frac{n_1}{n_0} = 3$) | LRT($\frac{n_1}{n_0} = 1$) | LRT($\frac{n_1}{n_0} = 3$) |
|---|---|---|---|---|---|
| 1.0 | 1 | 0.063 | 0.063 | 0.056 | 0.087 |
| 1.0 | 3 | 0.054 | 0.043 | 0.059 | 0.073 |
| 1.0 | 5 | 0.062 | 0.056 | 0.042 | 0.081 |
| 0.8 | 1 | 0.066 | 0.076 | 0.107 | 0.123 |
| 0.8 | 3 | 0.084 | 0.084 | 0.129 | 0.165 |
| 0.8 | 5 | 0.094 | 0.092 | 0.149 | 0.152 |
| 0.6 | 1 | 0.114 | 0.113 | 0.251 | 0.201 |
| 0.6 | 3 | 0.221 | 0.203 | 0.343 | 0.335 |
| 0.6 | 5 | 0.249 | 0.203 | 0.355 | 0.313 |
| 0.4 | 1 | 0.242 | 0.228 | 0.412 | 0.379 |
| 0.4 | 3 | 0.431 | 0.374 | 0.605 | 0.486 |
| 0.4 | 5 | 0.518 | 0.429 | 0.682 | 0.593 |
| 0.2 | 1 | 0.412 | 0.366 | 0.672 | 0.596 |
| 0.2 | 3 | 0.769 | 0.718 | 0.882 | 0.807 |
| 0.2 | 5 | 0.869 | 0.798 | 0.947 | 0.882 |

Table S6: Power analysis for RLD. The results are based on simulations with $N = 16, \rho = 0, p_0 = 0.5$.

| $\frac{p_1}{p_0}$ | $c_{max}$ | Log-rank($\frac{n_1}{n_0}=1$) | Log-rank($\frac{n_1}{n_0}=3$) | LRT($\frac{n_1}{n_0}=1$) | LRT($\frac{n_1}{n_0}=3$) |
|---|---|---|---|---|---|
| 1.0 | 1 | 0.038 | 0.033 | 0.111 | 0.128 |
| 1.0 | 3 | 0.039 | 0.034 | 0.067 | 0.059 |
| 1.0 | 5 | 0.037 | 0.042 | 0.076 | 0.063 |
| 0.8 | 1 | 0.110 | 0.053 | 0.234 | 0.243 |
| 0.8 | 3 | 0.117 | 0.048 | 0.224 | 0.219 |
| 0.8 | 5 | 0.090 | 0.058 | 0.237 | 0.250 |
| 0.6 | 1 | 0.267 | 0.171 | 0.404 | 0.345 |
| 0.6 | 3 | 0.298 | 0.201 | 0.501 | 0.434 |
| 0.6 | 5 | 0.304 | 0.201 | 0.484 | 0.435 |
| 0.4 | 1 | 0.428 | 0.375 | 0.575 | 0.549 |
| 0.4 | 3 | 0.653 | 0.511 | 0.792 | 0.716 |
| 0.4 | 5 | 0.633 | 0.513 | 0.832 | 0.746 |
| 0.2 | 1 | 0.732 | 0.649 | 0.845 | 0.801 |
| 0.2 | 3 | 0.936 | 0.869 | 0.985 | 0.944 |
| 0.2 | 5 | 0.964 | 0.885 | 0.992 | 0.975 |

Table S7: Power analysis for RLD. The results are based on simulations with $N = 16, \rho = 0, p_0 = 0.75$.

| $\frac{p_1}{p_0}$ | Log-rank($\frac{n_1}{n_0}=1$) | Log-rank($\frac{n_1}{n_0}=3$) | LRT($\frac{n_1}{n_0}=1$) | LRT($\frac{n_1}{n_0}=3$) |
|---|---|---|---|---|
| 1.0 | 0.000 | 0.000 | 0.000 | 0.000 |
| 0.8 | 0.053 | 0.004 | 0.518 | 0.189 |
| 0.6 | 0.398 | 0.171 | 0.892 | 0.773 |
| 0.4 | 0.834 | 0.659 | 0.994 | 0.978 |
| 0.2 | 0.985 | 0.984 | 1.000 | 1.000 |

Table S8: Power analysis for SHD. The results are based on simulations with $N = 16, \rho = 0, p_0 = 1$.

| $\frac{p_1}{p_0}$ | $c_{max}$ | Log-rank($\frac{n_1}{n_0}=1$) | Log-rank($\frac{n_1}{n_0}=3$) | LRT($\frac{n_1}{n_0}=1$) | LRT($\frac{n_1}{n_0}=3$) |
|---|---|---|---|---|---|
| 1.0 | 1 | 0.047 | 0.048 | 0.059 | 0.064 |
| 1.0 | 3 | 0.057 | 0.055 | 0.041 | 0.053 |
| 1.0 | 5 | 0.044 | 0.042 | 0.044 | 0.043 |
| 0.8 | 1 | 0.073 | 0.070 | 0.102 | 0.091 |
| 0.8 | 3 | 0.082 | 0.092 | 0.133 | 0.128 |
| 0.8 | 5 | 0.106 | 0.094 | 0.177 | 0.144 |
| 0.6 | 1 | 0.112 | 0.107 | 0.191 | 0.168 |
| 0.6 | 3 | 0.229 | 0.236 | 0.344 | 0.300 |
| 0.6 | 5 | 0.324 | 0.266 | 0.457 | 0.347 |
| 0.4 | 1 | 0.242 | 0.228 | 0.387 | 0.326 |
| 0.4 | 3 | 0.559 | 0.503 | 0.684 | 0.590 |
| 0.4 | 5 | 0.668 | 0.599 | 0.790 | 0.683 |
| 0.2 | 1 | 0.466 | 0.423 | 0.626 | 0.532 |
| 0.2 | 3 | 0.846 | 0.815 | 0.929 | 0.861 |
| 0.2 | 5 | 0.971 | 0.907 | 0.985 | 0.944 |

Table S9: Power analysis for RLD. The results are based on simulations with $N = 40, \rho = 0, p_0 = 0.25$.

| $\frac{p_1}{p_0}$ | $c_{max}$ | Log-rank($\frac{n_1}{n_0}=1$) | Log-rank($\frac{n_1}{n_0}=3$) | LRT($\frac{n_1}{n_0}=1$) | LRT($\frac{n_1}{n_0}=3$) |
|---|---|---|---|---|---|
| 1.0 | 1 | 0.043 | 0.057 | 0.044 | 0.050 |
| 1.0 | 3 | 0.060 | 0.065 | 0.058 | 0.075 |
| 1.0 | 5 | 0.044 | 0.055 | 0.061 | 0.060 |
| 0.8 | 1 | 0.082 | 0.090 | 0.140 | 0.167 |
| 0.8 | 3 | 0.154 | 0.124 | 0.236 | 0.190 |
| 0.8 | 5 | 0.141 | 0.111 | 0.227 | 0.216 |
| 0.6 | 1 | 0.230 | 0.227 | 0.367 | 0.349 |
| 0.6 | 3 | 0.424 | 0.378 | 0.572 | 0.505 |
| 0.6 | 5 | 0.494 | 0.395 | 0.636 | 0.522 |
| 0.4 | 1 | 0.531 | 0.456 | 0.665 | 0.574 |
| 0.4 | 3 | 0.837 | 0.748 | 0.905 | 0.827 |
| 0.4 | 5 | 0.898 | 0.801 | 0.957 | 0.883 |
| 0.2 | 1 | 0.837 | 0.791 | 0.906 | 0.856 |
| 0.2 | 3 | 0.989 | 0.983 | 0.997 | 0.995 |
| 0.2 | 5 | 1.000 | 0.993 | 1.000 | 1.000 |

Table S10: Power analysis for RLD. The results are based on simulations with $N = 40, \rho = 0, p_0 = 0.5$.

| $\frac{p_1}{p_0}$ | $c_{max}$ | Log-rank($\frac{n_1}{n_0}=1$) | Log-rank($\frac{n_1}{n_0}=3$) | LRT($\frac{n_1}{n_0}=1$) | LRT($\frac{n_1}{n_0}=3$) |
|---|---|---|---|---|---|
| 1.0 | 1 | 0.046 | 0.043 | 0.052 | 0.070 |
| 1.0 | 3 | 0.047 | 0.043 | 0.057 | 0.053 |
| 1.0 | 5 | 0.052 | 0.044 | 0.053 | 0.077 |
| 0.8 | 1 | 0.171 | 0.114 | 0.300 | 0.263 |
| 0.8 | 3 | 0.234 | 0.137 | 0.360 | 0.275 |
| 0.8 | 5 | 0.229 | 0.141 | 0.346 | 0.294 |
| 0.6 | 1 | 0.509 | 0.389 | 0.654 | 0.556 |
| 0.6 | 3 | 0.657 | 0.504 | 0.797 | 0.662 |
| 0.6 | 5 | 0.707 | 0.530 | 0.836 | 0.724 |
| 0.4 | 1 | 0.831 | 0.739 | 0.909 | 0.851 |
| 0.4 | 3 | 0.967 | 0.899 | 0.986 | 0.955 |
| 0.4 | 5 | 0.974 | 0.930 | 0.986 | 0.978 |
| 0.2 | 1 | 0.982 | 0.965 | 0.993 | 0.985 |
| 0.2 | 3 | 1.000 | 0.999 | 1.000 | 1.000 |
| 0.2 | 5 | 1.000 | 1.000 | 1.000 | 1.000 |

Table S11: Power analysis for RLD. The results are based on simulations with $N = 40, \rho = 0, p_0 = 0.75$.

| $\frac{p_1}{p_0}$ | Log-rank($\frac{n_1}{n_0}=1$) | Log-rank($\frac{n_1}{n_0}=3$) | LRT($\frac{n_1}{n_0}=1$) | LRT($\frac{n_1}{n_0}=3$) |
|---|---|---|---|---|
| 1.0 | 0.000 | 0.000 | 0.000 | 0.000 |
| 0.8 | 0.585 | 0.068 | 0.935 | 0.735 |
| 0.6 | 0.983 | 0.837 | 1.000 | 0.999 |
| 0.4 | 1.000 | 1.000 | 1.000 | 1.000 |
| 0.2 | 1.000 | 1.000 | 1.000 | 1.000 |

Table S12: Power analysis for SHD. The results are based on simulations with $N = 40, \rho = 0, p_0 = 1$

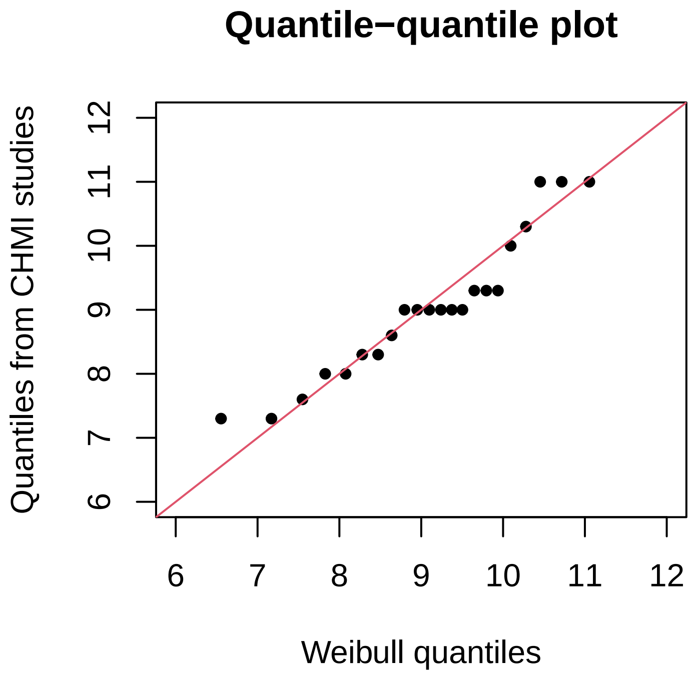


Figure S1: Quantile-quantile plot of time to infection from 5 previously published CHMI studies.

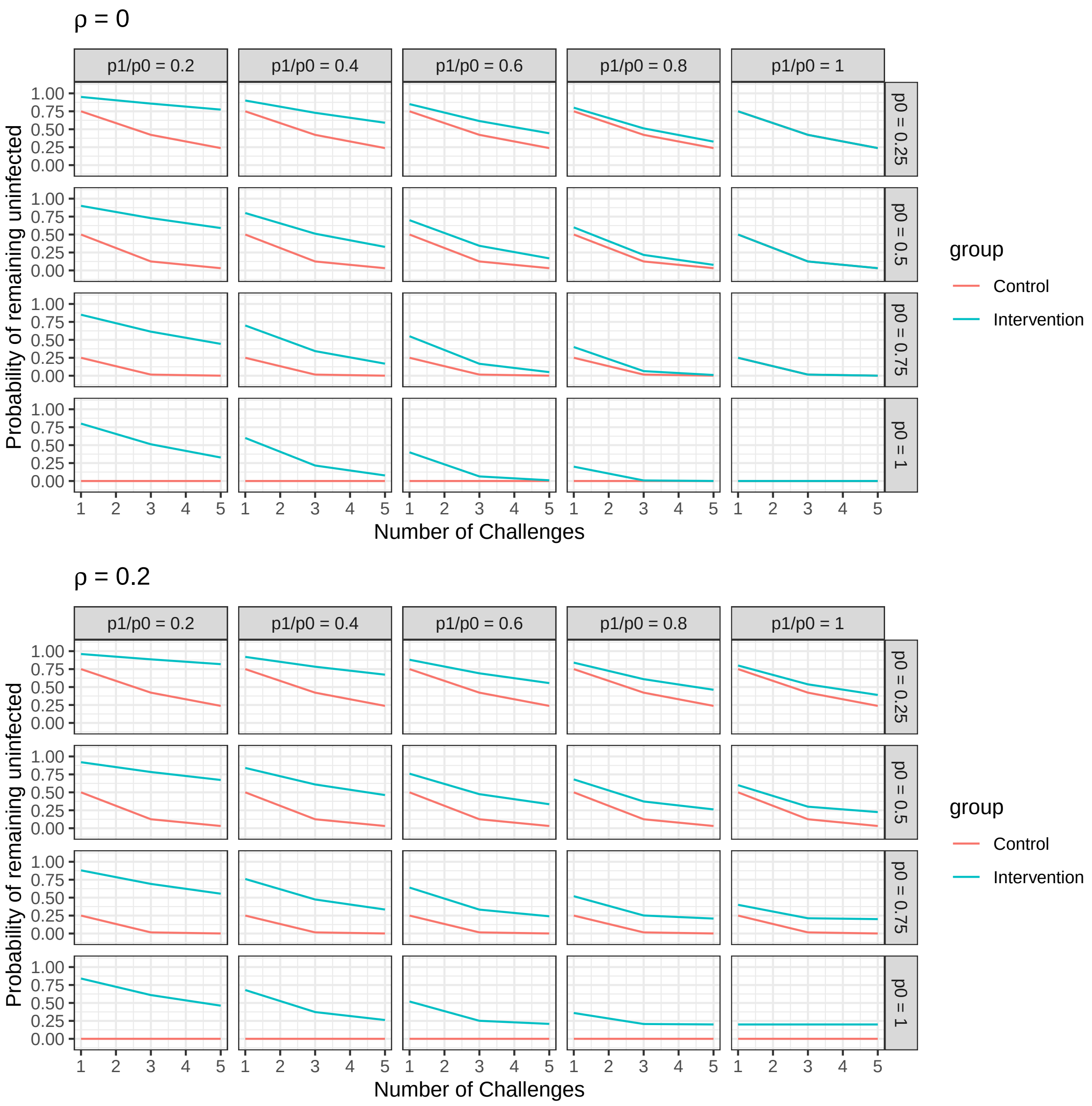


Figure S2: Probability of remaining uninfected against the number of challenges under leaky ($\rho = 0$) and mixture model ($\rho = 0.2$). $p_1$ and $p_0$ represent the probability of becoming infected after a single challenge in the intervention and control group, respectively.

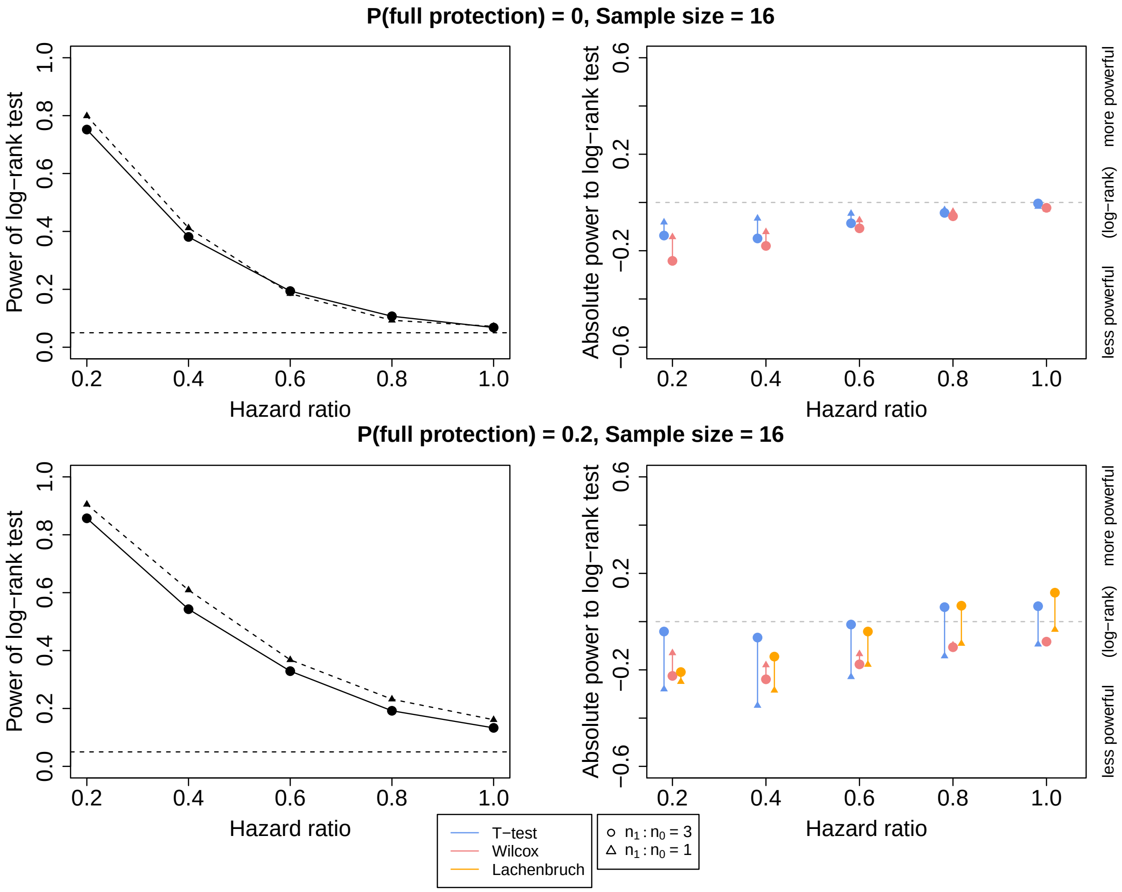


Figure S3: Power and type I error comparisons: Results are based on simulations with $N = 16$. The upper panels demonstrate the power of the log-rank test with different sample size allocations. Triangles represent simulations with $\frac{n_1}{n_0} = 1$ and circles represent simulations with $\frac{n_1}{n_0} = 3$. The lower panels demonstrate the absolute difference in power when comparing the t-test, Wilcoxon test, and Lachenbruch test to the log-rank test. The log-rank test is less powerful if the absolute power of the log-rank test is negative.

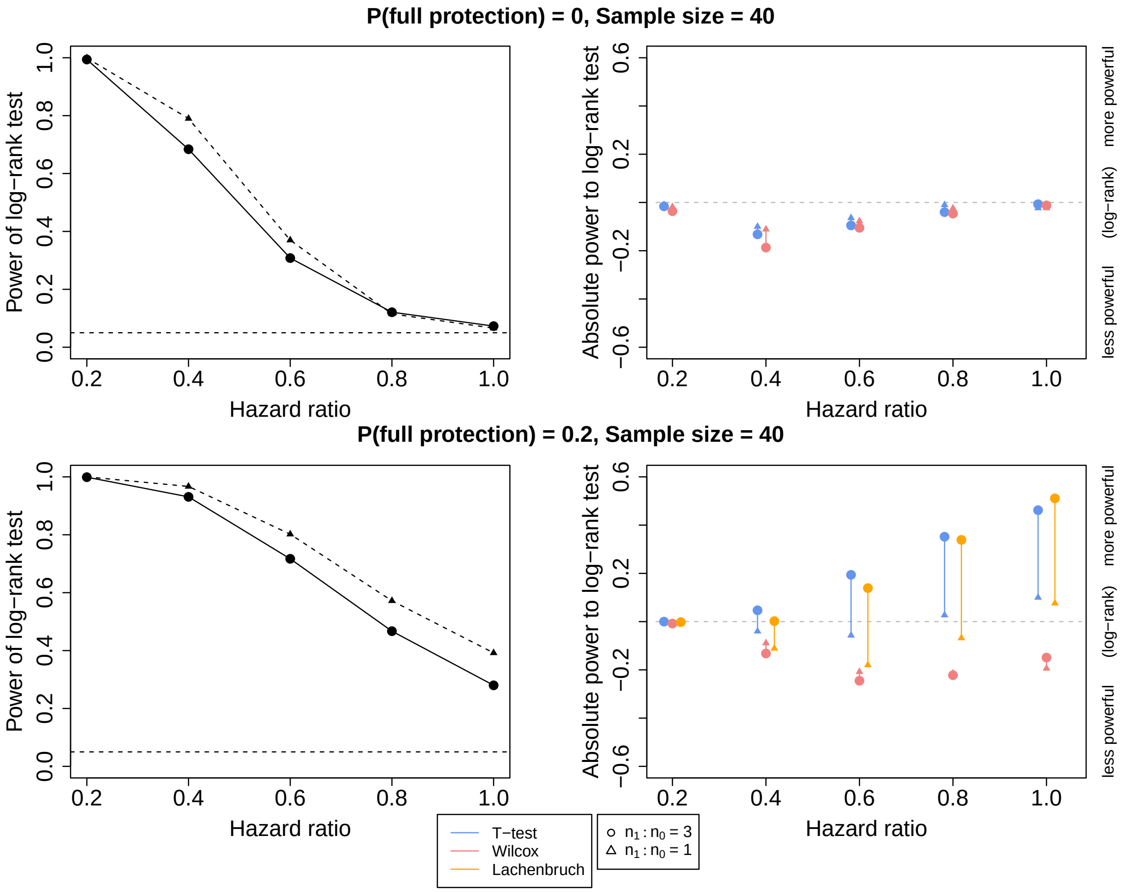


Figure S4: Power and type I error comparisons: Results are based on simulations with $N = 40$. The upper panels demonstrate the power of the log-rank test with different sample size allocations. Triangles represent simulations with $\frac{n_1}{n_0} = 1$ and circles represent simulations with $\frac{n_1}{n_0} = 3$. The lower panels demonstrate the absolute difference in power when comparing the t-test, Wilcoxon test, and Lachenbruch test to the log-rank test. The log-rank test is less powerful if the absolute power of the log-rank test is negative.

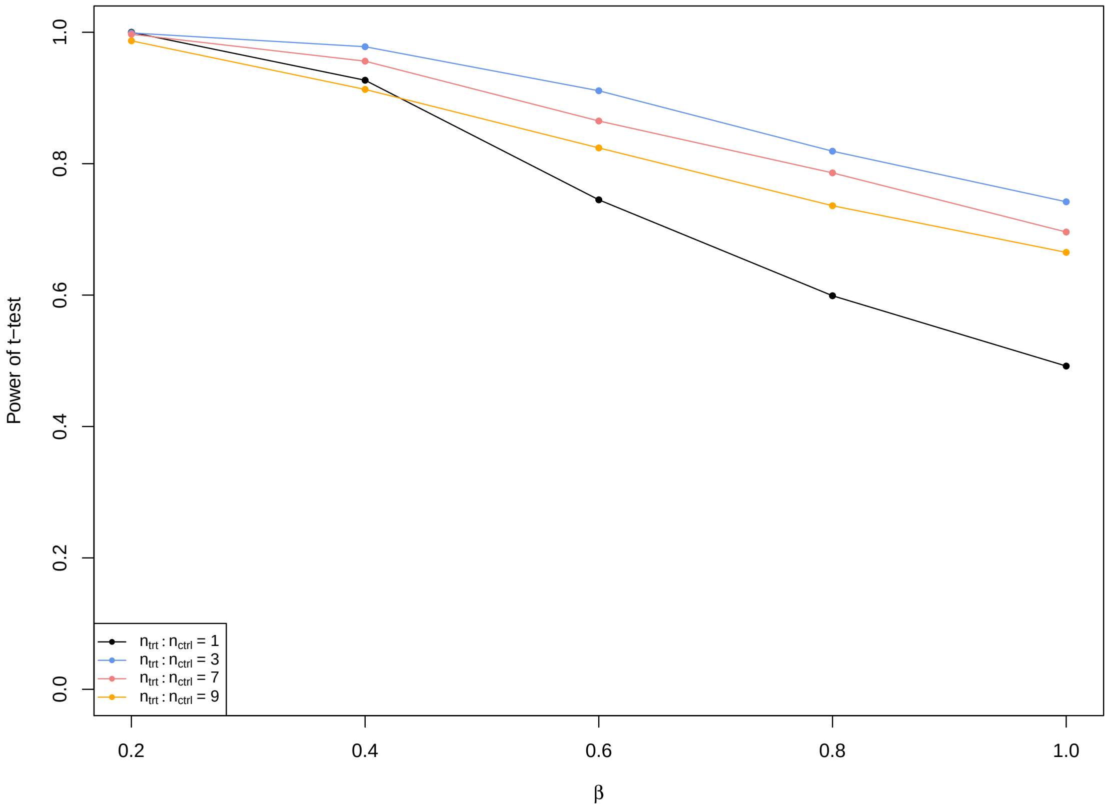


Figure S5: Power of the t-test for different sample size allocations: Results are based on simulations with $\rho = 0.2$. The t-test is most powerful when $\frac{n_1}{n_0} = 3$. The square root of the ratio of variance between the intervention and control groups in the simulated data ranges from 4.2 to 4.6 for $\beta \in [0.2, 1]$.

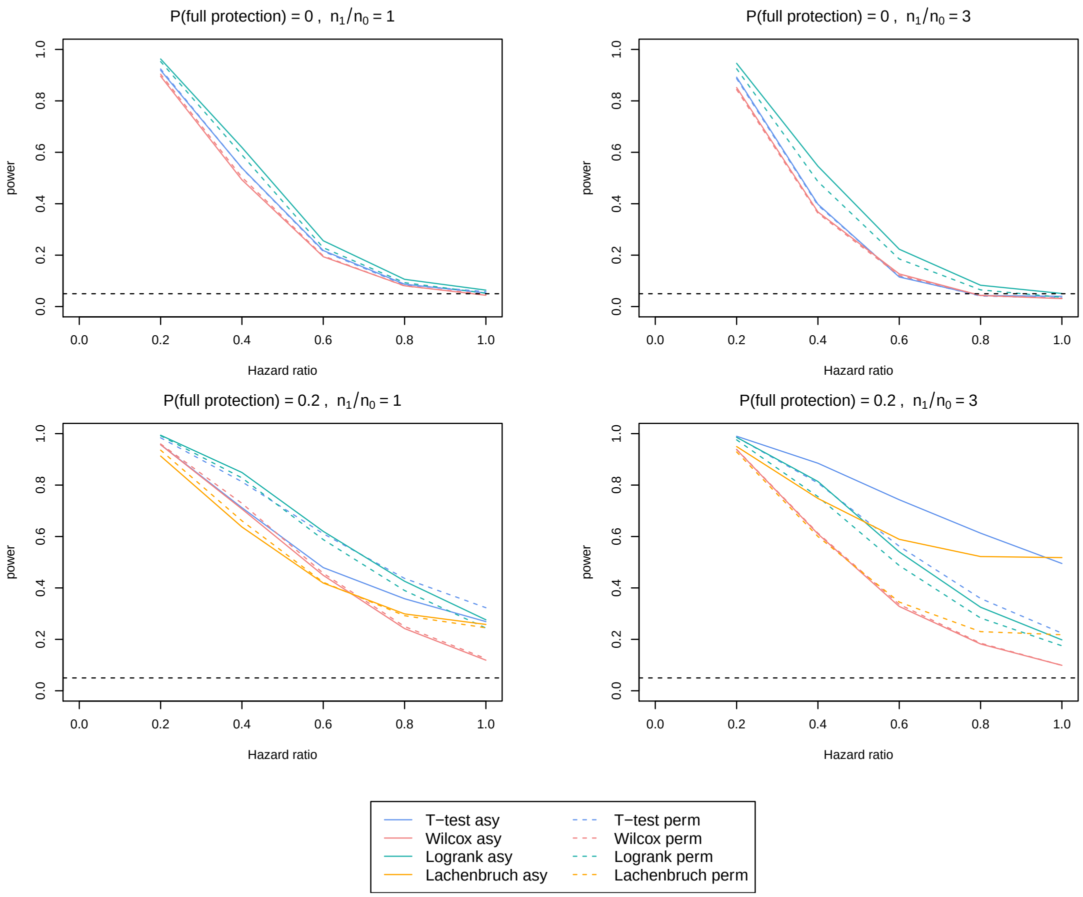


Figure S6: Power and type I error comparisons of asymptotic-based and permutation-based tests. Results are shown for different sample size allocations and for $\rho = 0$ and $\rho = 0.2$. The total sample size is fixed at 28.

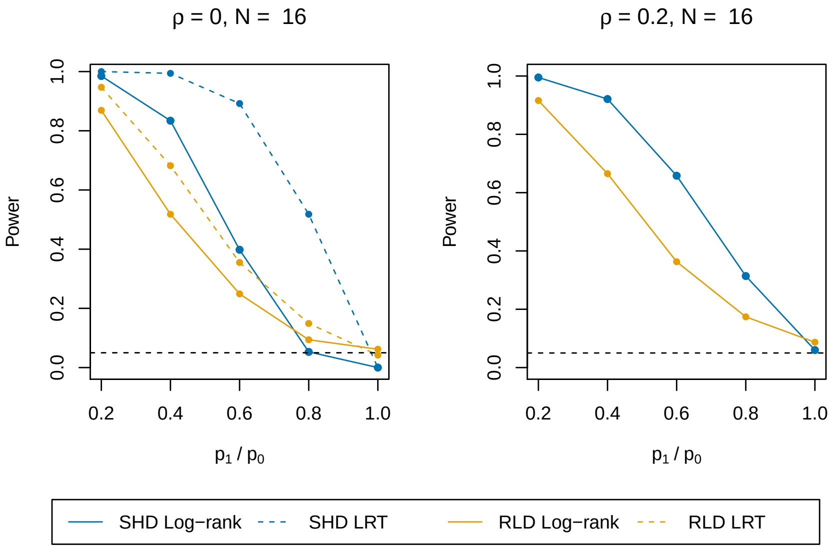


Figure S7: Power and type I error comparisons. The results are based on simulations with $n_0 = n_1 = 8, p_0 = 1$ for SHD, $p_0 = 0.5$ and $c_{max} = 5$ for RLD. With relative risk being 1, the points represent the type I error.

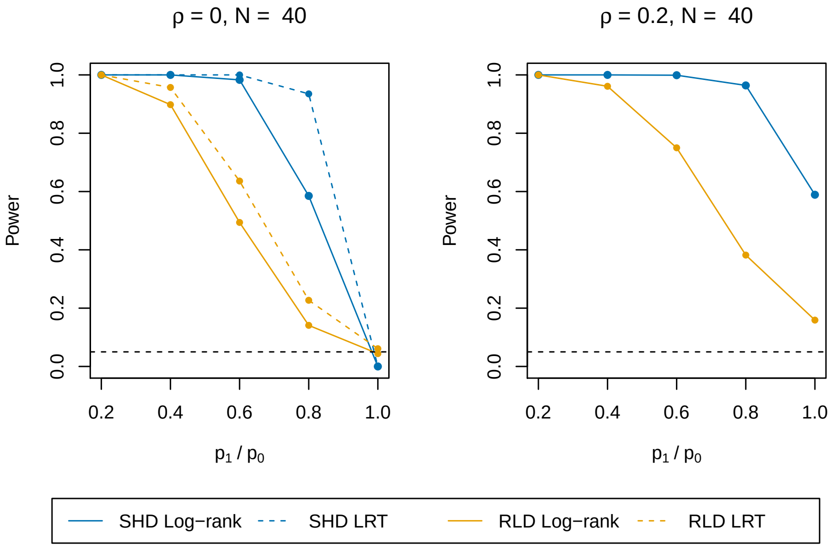


Figure S8: Power and type I error comparisons. The results are based on simulations with $n_0 = n_1 = 20, p_0 = 1$ for SHD, $p_0 = 0.5$ and $c_{max} = 5$ for RLD. With relative risk being 1, the points represent the type I error.

**Likelihood ratio test for continuous survival time model**

Throughout it is assumed that $n_0$ iid copies of $(T_{0i}, \delta_{0i})$ from the control group where $\delta_{0i}$ is an indicator variable for observing the event. Similarly, $n_1$ iid copies of $(T_{1i}, \delta_{1i})$ from the intervention group are observed.

Under the alternative hypothesis of mixed intervention effect, the contribution to the likelihood from the controls is $L_{ctrl} = \prod_i^{n_0} f(T_{0i}; k, \lambda_0)^{\delta_{0i}} S(T_{0i}; k, \lambda_0)^{1-\delta_{0i}}$ where $f$ and $S$ are the probability density and survival function for the Weibull distribution with parameters $k$ and $\lambda_0$ as described in the methods section. For intervention group the likelihood contribution is $L_{trt} = \prod_i^{n_1} [f(T_{1i}, k, \lambda_1)(1-\rho)]^{\delta_{1i}} [\rho + (1-\rho)S(T_{1i}, k, \lambda_1)]^{1-\delta_{1i}}$ where $\rho$ is the parameter for the Bernoulli variable indicating indicating whether the individual is fully protected. Standard numerical optimization software can be used to maximize the likelihood $L(k, \lambda_0, \lambda_1, \rho) = L_{ctrl} L_{trt}$.

Under the null hypothesis of no full protection, the likelihood under the the null hypothesis is $L_0(k, \lambda_0, \lambda_1) = L(k, \lambda_0, \lambda_1, \rho = 0)$ and likelihood ratio statistic follows a mixture of chi-squared distribution $\frac{1}{2}\chi_0^2 + \frac{1}{2}\chi_1^2$.

Under the null hypothesis of no delay of infection time, $\lambda_0 = \lambda_1$, the likelihood under the the null hypothesis is $L_0(k, \lambda_0, \rho) = L(k, \lambda_0, \lambda_1 = \lambda_0, \rho)$ and likelihood ratio statistic follows a mixture of chi-squared distribution $\frac{1}{2}\chi_0^2 + \frac{1}{2}\chi_1^2$ under the constraint that $\frac{\lambda_1}{\lambda_0} \leq 1$.

Under the null hypothesis of no intervention effect, the likelihood under the null hypothesis is $L_0(k, \lambda_0) = L(k, \lambda_0, \lambda_1 = \lambda_0, \rho = 0)$. The likelihood ratio statistic follows a mixture of chi-squared distributions with degrees of freedom 0, 1, and 2. In our simulation studies, it is found that nominal type I errors are better controlled using the empirically derived asymptotic distribution distribution of the likelihood ratio statistic [1] instead of the theoretical mixing probabilities [2] ($\frac{1}{4}\chi_0^2 + \frac{1}{2}\chi_1^2 + \frac{1}{4}\chi_2^2$).

**References**

1. Stoel R, Galindo-Garre F, Dolan C, Wittenboer G. On the likelihood ratio test in structural equation modeling when parameters are subject to boundary constraints. Psychol Methods. 2007;11:439–55.
2. Self SG, Liang KY. Asymptotic properties of maximum likelihood estimators and likelihood ratio tests under nonstandard conditions. J Am Stat Assoc. 1987;82:605–610.
